# Health care in a homophobic climate: the SPEND model for providing sexual health services to men who have sex with men where their health and human rights are compromised

**DOI:** 10.3402/gha.v8.26096

**Published:** 2015-03-17

**Authors:** Michael W. Ross, Joyce Nyoni, Markus Larsson, Jessie Mbwambo, Anette Agardh, John Kashiha, Sheryl A. McCurdy

**Affiliations:** 1 Program in Human Sexuality, Department of Family Medicine, University of Minnesota, Minneapolis, MN, USA; 2 Department of Clinical Sciences Malmö, Division of Social Medicine and Global Health, Faculty of Medicine, Lund University, Lund, Sweden; 3 Department of Sociology and Anthropology, University of Dar es Salaam, Dar es Salaam, Tanzania; 4 Department of Psychiatry and Mental Health, Muhimbili University of Health and Allied Sciences, Dar es Salaam, Tanzania; 5 Center for Health Promotion and Prevention Research, School of Public Health, University of Texas, Houston, TX, USA

**Keywords:** men who have sex with men, homosexual, discrimination, stigma, Africa, HIV, STIs, treatment

## Abstract

We present a model for developing health services for men who have sex with men (MSM) in sub-Saharan Africa and other places where MSM are heavily stigmatized and marginalized. The processes of the SPEND model include *S*afe treatment for sexually transmissible infections (STIs) and HIV; *P*harmacy sites for treatment of STIs in countries where pharmacies and drug stores are the source of medical advice and treatment; *E*ducation in sexual health issues for health professionals to reduce discrimination against MSM patients; *N*avigation for patients who have HIV and are rejected or discriminated against for treatment; and *D*iscrimination reduction through educating potential leaders in tertiary education in issues of human sexuality. Supporting empirical evidence from qualitative and quantitative studies is summarized, and barriers to implementation are discussed. Health care for MSM is one of the casualties of anti-homosexual social and legal climates. There is no amnesty for MSM in health care settings, where the stigma and discrimination that they face in the rest of society is replicated. Such conditions, however, make it necessary to consider ways of providing access to health care for MSM, especially where rates of HIV and STIs in MSM populations are high, and stigma and discrimination encourages high proportions of MSM to marry. This in itself enhances the status of MSM as an important bridge population for STIs including HIV. Where anti-homosexual laws encourage, or are believed to encourage, the reporting of MSM to authorities, health care may be seen as an agent of authority rather than an agency for care.

The Ugandan Anti-Homosexuality Act of 2014 (1, 2) provided for penalties of up to life imprisonment for gay sex, and lesser penalties for supporting LGBT (lesbian, gay, bisexual and transgender) rights, and Ugandans overseas could be extradited back to Uganda for punishment. This was nullified by the Ugandan Supreme Court but is about to be reintroduced into parliament. In January 2014, a Nigerian bill criminalized ‘a person or group of persons who supports the registration, operation and sustenance of gay clubs, societies, organizations, processions or meetings’, (3) and there are reports of men who have sex with men (MSM) in Nigeria being tortured to reveal the names of gay acquaintances, and of public whippings of gay men. In Gambia, President Jammeh enacted an amendment to existing criminal codes against homosexuality, in October 2014, making ‘aggravated homosexuality’ conviction a life-time sentence. Most countries in sub-Saharan Africa (SSA) with British colonial legal bases (South Africa, which provides for equality before the law for sexual orientation, is a notable exception) currently have laws providing for up to 14 years imprisonment for homosexual behavior but several have more draconian laws (based on the Ugandan law) foreshadowed (4). Nor is this limited to Africa: in Russia, President Putin signed into law in June 2013 a prohibition on discussion or support of homosexuality (5). The effect of these laws is to produce a climate which encourages violence and abuse against those who are or are suspected of being gay or lesbian and which reinforces stigmatization of LGBT individuals by health personnel. Such legislative and social discrimination, which may include provisions for punishing health care personnel who do not inform on patients, make it difficult to provide services for those known or suspected of gay or lesbian behavior or activities or do not conform with gender normativity. Although provisions to force health practitioners to inform on homosexual patients were dropped from the bills in Nigeria and Uganda, many MSM believe that they are part of the law, and distrust disclosure to health workers: there are reports that attendance of MSM at health centers is dramatically reduced (6). Even where there is equality before the law, a homophobic climate may encourage such violations as ‘corrective rape’ and murder of lesbians, and discrimination and abuse against gay and lesbian patients have been well documented in South Africa (7, 8).

Findings from several studies stand in contrast to the commonly placed argument by sub-Saharan African governments that homosexual and bisexual men and women either do not exist, are ‘unAfrican’, or only constitute a small number of individuals ‘corrupted’ by Western standards (9–11). This has motivated underfunding of targeted health programs, and punitive laws (12).

In a cross-sectional study targeting Ugandan university students, 25% of the females and 15% of the males in 2005 reported having had sexually fantasies about someone of the same sex and 16% of the females and 8% of the males having had sexual relations with someone of the same sex; in 2010, the same figures were 20% for females and 10% for males, respectively 10% for females and 6% for males (13). In his study from Botswana, McAllister (14) investigated the emergence of a local gay culture among LGBT populations and observes that a process of localizing gay identities (he calls it ‘Tswanarisation’) is taking place. This process of developing a Setswana way of being gay, and as such emancipate from the Western gay culture, is an important observation as several leaders and power holders continue to claim that homosexuality is ‘unAfrican’. Similar observations have been made elsewhere in Africa, for example in Uganda where activists have constructed names for gay and lesbian in their native language (15).

For many who work in such hostile health climates overseas, and local professionals working in such climates, there is a sense of unease and sometimes helplessness about providing sexual health services to sexual minorities. Beyrer et al. (16) note that MSM are often excluded, sometimes systematically, from HIV services because of stigma and that in many settings, they do not have access to the most basic of HIV services (including testing, counseling, and condoms and lubricants). Exposure to discrimination and stigma has severe implications for the well-being of LGBTs. A study from Kampala, Uganda, found that MSM who were subjected to homophobic abuse were five times as likely to be HIV infected compared to those unexposed (17). In Kenya, Geibel et al. (18) saw associations between discrimination and inconsistent or no condom use among MSM, while in Tanzania Anderson et al. (19) found high levels of abuse and discrimination against MSM: those experiencing high discrimination and violence had a significantly greater number of sexual partners, depression scores, and internalized homonegativity (IH) scores. High IH in turn predicted HIV infection.

While we recognize that stigmatization is an issue for LGBT populations generally, we focus on HIV and sexually transmissible infections (STIs) in MSM. HIV in MSM frequently occurs in environments that are not just resource-constrained but actively hostile to MSM. While Beyrer et al. (16) provide a far-reaching analysis and description of the HIV services required for MSM and barriers to achieving them, Smith et al. (12) also confirm the urgent need to immediately develop and implement appropriate HIV interventions for MSM, which will require a range of different service delivery models to meet the unique biological and particularly social conditions that put MSM at increased risk. We provide such a model, the SPEND model, for addressing some of the more proximal services and barriers for HIV/STIs in MSM in SSA taking into account the pervasive political, cultural, social, and economic stigma.

The model is based first on the provision of health services and, second, on reducing the discrimination that inhibits the provision of such services, starting with the most immediate health needs and moving to more distal discrimination. SPEND is the acronym for (S) Safe treatment; (P) Pharmacies as treatment sources; (E) Educate health professionals; (N) Navigation for patients who must access the health system; and (D) Discrimination reduction. The most crucial elements of the model are the ones most proximal to health services and hence more likely to reduce disease: Safe treatment, when HIV/STIs are identified, Navigation of patients to health services where they will not be denied treatment, and Pharmacies for treatment. More distal are the longer-term issues which attempt to change the hostile climate, specifically Education for health professionals, and Discrimination reduction through general education of future leaders.

We cite a mixture of empirical studies already published, and some qualitative interview data from our Tanzanian MSM study (20). Briefly, for the qualitative arm of the study, we interviewed 300 MSM in Dar es Salaam (*n*=200) and Tanga (*n*=100) using Respondent-Driven Sampling, and carried out an in-depth qualitative interview in Swahili with every tenth participant. Transcribed interviews were analyzed by themes by ML, and the transcripts read independently by MR, AA and JN and the themes discussed and further modified.^1^ The study was approved by both the Tanzanian National Institute for Medical Research and the University of Texas IRBs.

## Safe treatment

The *Safe treatment* component is based on the need to provide screening and treatment for MSM who are excluded, either by discrimination or the expectation of discrimination, from sexual health services (this should include diagnosis and treatment of anal and oral STIs). Ideally, in the longer term, this will include full access to treatment in the available STI clinics with more trained, skilled and accommodative staff who would offer services without prejudice and stigmatizing their clients. Until then, in the short term, treatment needs to be accepting so as to be safe psychologically, and safe in terms of being in a non-identifiable place. Locations which are identified as being specifically for MSM may draw media exposure, hostile crowds, and risk of vigilante activity. Several sites need to be identified and as soon as one becomes known, there should be another to take its place. Mixed sites where other populations are treated are ideal as it is important to have ‘plausible deniability’ whenever possible for MSM attending, and rotating screening associated with MSM venues is likely to have the greatest impact on disease (especially if it is for all males regardless of sexual orientation, given that most MSM venues are mixed).

Empirical evidence supporting this approach includes that of 300 MSM surveyed by Ross et al. (20) in Tanzania. In Dar es Salaam, 30% tested had HIV infection (only five were previously aware of infection) and nearly 20% had either (or both) gonorrhea or *Chlamydia* infection, almost all of it rectally. Yet because of high reported discrimination and abuse in STI and other treatment facilities, almost all the men would not attend or if they did, reported heterosexual activity as the source of their infection. Interviewee MSM-5 observed that ‘… if the problem is at the anus, I have to tell them that the problem is at my penis’.

We have surveyed gay and MSM-related sites in Dar es Salaam (21) and noted 98 sites, evenly distributed throughout the metropolitan area with no geographic focus. If typical for larger SSA cities, as we believe it is, our mapping indicates gay-associated sites are widely distributed throughout the metropolitan areas and probably serve local populations. Those which are amenable to acting as treatment sites include several dance halls and discos with weekend-only gay emphasis, and restaurant/bars where a small back room could be used for testing. The viability of sites may frequently be threatened when their presence becomes known. Kajubi et al. (22) attempted to recruit 500 MSM in Kampala but were forced to stop the study after recruiting only 224 when a national newspaper publicized the study, including the recruiting coupon which would identify the holder as gay. In Mombasa, demonstrations were organized against the Kenya Medical Research Institute (KEMRI) research center in Kilifi, a center for HIV/AIDS prevention and treatment, and later calling for the closure of KEMRI: ‘how can a state institution be involved on the pretext of providing counseling services to these criminals?’ (23).

However, concerns about lack of professional confidentiality and fear of exposure remain central and justifiable concerns for MSM who interface with health professionals. In the qualitative component of our Dar es Salaam study (20), interviewee MSM-8 stated that ‘I once had a STD then I went to the hospital and tell the doctor about my problem. Before I left the hospital that doctor went out and tell other doctors that I am gay’. Interviewer: So you were not given service? MSM-8: ‘No, I was not given any service’. Even worse may be anticipated, as Respondent MSM-10 explained: ‘I went to [Hospital name] and I was wearing makeup so the doctor told me that I am gay so I am not allowed to get treatment from that hospital. He told me to leave as soon as I can because he would inject me with poison, I was really hurt and I left without being treated ….’

Privacy is a crucial component of care for stigmatized patients and can be highly variable even within cities: Kagashe and Rwebangila (24) note that even for (presumed heterosexual) patients in Dar es Salaam, there is high variation between clinics on privacy and very different attitudes to care received. Generally, private institutions are better regarded than government services, although private services may not be available to poorer patients. Interviewee MSM-12 noted that ‘Things are different in the private hospitals as they respect us, but in government hospitals they do not care at all, even if you die they see you as a dog’ and ‘Gays that can afford private hospitals have no problems but for poor ones like me things are not easy at all’.

Barriers to ‘Safe treatment’ include obstruction of services for sexual minority populations, lobbying against services for sexual minority populations by religious movements, and the reluctance of MSM who do not identify as gay or bisexual to undertake screening which may imply MSM activity. Challenges include identifying sites of MSM meeting places, identifying and renting unidentified places adjacent for screening, taking anal swabs from men who may be afraid it will identify their sexual behaviors, the fact that many known sites cater to a subculture which is focused on younger MSM, identifying a shop front that has ‘plausible deniability’ for attending, being able to rapidly change sites when they become compromised, and potential vigilante activity.

## Pharmacies as treatment sources

The *Pharmacy* component is based on the finding that because of stigma and discrimination in health care facilities, treatment for sexually related (including HIV) concerns is in SSA in the first instance (and usually thereafter) sought from pharmacies and drugstores due to accessibility, lower costs and a perception that there is greater privacy (25, 26). Among marginalized populations such as MSM, public health settings and hospitals are also associated with actual and/or perceived discrimination. This is due to discrimination and abuse (or perceived discrimination) in public health care settings: as respondent MSM-1 commented, ‘… you feel it's better to go to the nearest pharmacy and buy medicine rather than going to the health center … going to the hospital will lead to stigmatization from doctors and nurses’. Private clinics are acknowledged to be much less discriminatory, but their cost is prohibitive to all but the affluent. However, pharmacies and drugstores are likely to offer inappropriate antibiotics and in inappropriate doses, or not provide appropriate testing. Our interviewee MSM-6 said that he ‘… started by going to dispensaries but they failed to test me, so I went to [Hospital name] where I was treated’.

Empirically, Viberg et al. (27) used simulated clients who attended requesting STI medications in drugstores in Tanzania and found that while three quarters of drugstores said they did not have STI medications, for 78% of males and 63% of females, drugs were dispensed: for 80% of those males and 90% of those females, these drugs were those recommended for syndromic management of STIs. Few of the drugs dispensed were totally irrelevant but dosage regimens were often incorrect and complete syndromic management was rarely provided. During simulated visits to pharmacies in Kenya, Mugo et al. (28) found that only 10% prescribed recommended antibiotics at recommended dosages and duration, However, Viberg et al. (27) note that in some countries in SSA, pharmacies are the preferred healthcare provider for STI patients, and that private drugstores should be given a more formal role in STI management. Viberg et al. (29) subsequently report that drug-sellers have considerable practical knowledge of antibiotics. Our unpublished MSM data from Tanzania indicate that between a fifth and a sixth of MSM go to a pharmacy or drugstore for STI symptom treatment, while depending on the condition up to one in two will self-medicate. Such an approach may not be ideal for rectal STIs, given their low level of symptoms, so pharmacies should not be regarded as a full substitute for adequate medical assessment and treatment. On the other hand, some MSM report that they are prescribed multivitamins or analgesics when requesting treatment for STIs (it is unclear if this is for urethral or anal symptoms) in public clinics, so it is uncertain the level of appropriate treatment that is given even in medical facilities.

Barriers to identifying and utilizing ‘Pharmacies as treatment sources’ may be enforcement of local laws prohibiting providing antibiotics without prescription, the cost of medications, possible medical opposition to what may be seen as an encroachment on medical practice, and negative attitudes to sexual minorities among pharmacy and drug store staff. Challenges include identifying willing pharmacies, training the staff, and staying in compliance with local laws regarding what pharmacies and drugstores may dispense without prescription.

## Education for health professionals

The third component involves *Education for health professionals*. Based on the extensive experience of the authors in East Africa, there is widespread misinformation about human sexuality which is used to justify discrimination. While key issues for nursing sexuality education in Western systems involve knowledge, skills, attitudes toward sexuality, opinions about professional role, comfort with sexuality and continuing education in the area (30), data from East Africa suggest that nurses disapprove of adolescent sexual activity, including masturbation (which they thought dangerous), contraceptive use and abortion, and had generally very conservative attitudes toward sexual and reproductive health issues (31). Those with more education including those who had continuing education on sexuality tended to have more liberal attitudes. In a study of why nurses abuse patients in South Africa, Jewkes, Abrahams and Mvo (32) report that humiliation and abuse of patients by nurses is both reactive and ritualized. Studying patient abuse by nurses in obstetric services, they note that the practice is widespread and related to professional insecurities and need to assert control and power, plus lack of corrective action from supervisors and managers. Jewkes et al.'s data indicate that abuse of patients is far more widespread than just with MSM and that sexuality training plus the broader context of nurse–patient relationships need to be addressed. Tanzanian data on lack of interpersonal skills in health personnel and discriminatory and judgmental treatment in primary health care services (33) support these findings. Our interviewee MSM-2 said ‘Train them [health care workers] so that they can like gays, train them [to treat them] just like other human beings instead of pointing fingers at them’. Interviewee MSM-12 made a similar comment: ‘… they have to stop staring at us and say that we don't know about God while we know Him very well’. Significantly, data from Kenya suggest that health professionals themselves need and want training to better understand the situation and needs of MSM in order to better serve and counsel them (34).

Barriers for ‘Education for health professionals’ include negative attitudes and beliefs about sexual minorities in academics in the health professions and consequently persuading nursing and medical schools (some of which may be church-sponsored schools) to allow such workshops, and challenges include making the classes a regular part of the curriculum rather than voluntary.

## Navigation for patients

The fourth component for MSM health is *Navigation*. MSM with health conditions requiring treatment (most commonly STIs and HIV disease) in our Tanzanian studies report that active discrimination prevents them from attending for treatment, including being ignored by nursing staff and reception staff once it is known that they are gay or bisexual, and including frank rejection (such as telling MSM that they deserve the disease and won't get treatment). As interviewee MSM-11 commented, ‘Once you get to the hospital and they happen to know that you are gay all they do is call each other and stare at you instead of helping you’. Respondent MSM-2 told us ‘I went to a hospital 1 day and a doctor learned that I am gay and he told other doctors that I am gay. There is a doctor who was supposed to treat me but he didn't help me, instead he treated another woman who came to hospital later than me’. This may occur in countries despite provision of HAART through foreign donation and in-country health plans. Even if reports are exaggerated, there is a common belief in the MSM community that such discrimination regularly occurs.

The concept of Patient Navigator (PN) was originally developed in cancer care (35) to help patients through the maze of treatment, including temporal (helping keep appointments); geographic (helping steer patients through large medical centers); administrative (assisting with filling in paperwork); transportation (getting to and from appointments); and in linkages (referrals to other medical and social services). We propose that PNs add to these roles protection against discrimination, particularly the discrimination and abuse that is described as being common to all patients by studies in the primary health system in several SSA countries. PNs have been described in HIV care (36) and have achieved reduction in barriers and improved health outcomes for underserved populations with HIV disease in the U.S. These positive outcomes were achieved through reduction of structural barriers and improved provider engagement. In SSA, PNs who are nurses should accompany MSM with HIV positive test results to make and subsequently keep appointments to prevent them being denied treatment or abused, and to carry out the other tasks of navigating the health system that are already associated with PNs.

Barriers for ‘Navigation for patients’ include the relative loss of anonymity for the patients, and opposition from health care facilities to allow Navigators to accompany patients. Challenges include recruiting empathic health workers.

## Discrimination reduction

The final component, *Discrimination reduction*, is a long-term goal but essential to provide a social and human rights climate to enable MSM to access health care without fear of discrimination. Human sexuality education courses for non-health professionals should be made available, preferably as part of tertiary education programs. We base this on the principle that discrimination against MSM in East Africa that is enshrined in legislation and accepted by social leaders can only be reduced, in the long term, by a fuller understanding of human sexuality based on empirical and human rights data. While there are empirical data in Western countries that note differences between men and women in attitudes toward gay men, but no gender differences in the way they view gay civil rights (37), high religiosity plays a major part in negative attitudes toward homosexuality especially the attitudes of women (38). Nonetheless, the belief that homosexuality is biologically based and fixed early in life seems to be the most important mediator in antigay prejudice independent of conservatism and authoritarianism (39). The deficit of studies of attitudes toward sexuality and antigay prejudice in Africa, however, makes generalization of Western studies dubious.

In the longer term, clinicians and researchers also need to consider *restoration services* that will restore the mental health, self-efficacy and sense of self-worth to gay and bisexual men, especially given the findings that IH has serious mental health and HIV risk behavior implications (40, 41). Credible community outreach must involve local LGBT NGOs. Repression of emergent community-based organizations that suffer government harassment, suppression or de-registration is often unintentionally facilitated by Western organizations that require large and viable NGO partners with long-term histories of collaboration.

Barriers to ‘Discrimination reduction’ include existing negative attitudes toward sexual minorities, which mitigate against being able to convince tertiary institutions to include such courses, preventing students who attend them being stigmatized, and preventing opposition from extremist religious groups. Challenges include the fact that such classes will be voluntary, getting sponsorship to allow students to attend or cover fees, and provision of credit hours.

We provide the SPEND model as a proximal prototype to begin to conceptualize the steps that can be taken to introduce programs that begin to address provision of sexual health services to MSM in hostile climates. In some countries, many of these issues have been recognized and steps taken to introduce approaches that deal sensitively with stigmatized key populations (42). While we have based this model on our work in SSA, we believe that it may have utility in other similar environments including the Middle East and the former Soviet Union. It is a short-term approach to high levels of STIs (including HIV) in MSM populations and will hopefully become unnecessary as discrimination and stigma associated with homosexual behavior are removed from health care practice.

## Footnotes

1 Larsson M, Ross MW, Nyoni J, Månsson SA, Shio J, Agardh A. Being forced to become your own doctor—a qualitative analysis of men who have sex with men's experiences of stigma in the Tanzanian health care system. In submission.
